# Brief Reviews for the Institutional Reader

**Published:** 1913-09-20

**Authors:** 


### BRIEF REVIEWS FOR THE INSTITUTIONAL READER.

Disinfection and Disinfectants. By M. Christian. Translated from the German by C. Salter. (London: Scott, Greenwood and Son. 1913. Price 5s. net.)

This translation of a German work on a very practical subject is a little disappointing. The book does not appear to be of such practical value to English public health authorities as to warrant its publication in this country. It might reasonably have been expected that after all the trouble and care spent in producing such a manual there would have been found in it a more thorough and a more practical treatment of this important subject. As it is, the book is useful enough, but its practical value, to a very great extent, remains Teutonic. The principle of such machines as the Washington Lyons is inadequately dealt with, but some useful matter is to be found on the subjects of formalin vapour machines and on chemical disinfectants. Many of the illustrations are very poor and not sufficiently instructive, while the price of the book seems to be unnecessarily high.

Provincial Hospital Pharmacopœias. Comprising the Formulas for Medicinal Preparations used in Twenty-five Hospitals and Infirmaries in Great Britain. (London: The Office of *The Chemist and Druggist.* 1913. Pp. 288. Price 2s. 6d.)

National Insurance Prescriptions for the Use of Medical Practitioners. (Edinburgh: *The Prescriber* Offices. 1913. Price 6d.)

One effect of the National Insurance Act has been the stimulation of the dormant facility of prescription-writing among medical men. But there has been a marked tendency to return to the old methods of stock mixtures, most of which are founded upon the prescriptions in some favourite hospital Pharmacopœia. This method may be euphemistically called encouraging standardisation, but is probably much safer than the dangerous originality of the inexperienced prescriber. The first of the two little books now before us offers an almost overwhelming choice of the ready-made prescription. It consists of the formulas used in twenty-five provincial hospitals. As a painstaking collection of well-tried recipes and also of pharmaceutical fatuities, the book possesses some usefulness and interest. It is a matter for marvel to observe the number of variations that can be made with our old friends Mist. Alba. and Mist. Gent. Alk. Why it should be considered necessary to conglomerate these hundreds of prescriptions of purely local interest it is difficult to understand. The other work is a tiny pamphlet of thirty pages, containing prescriptions for the use of panel doctors. It is hardly credible that any but the most ignorant practitioners would find this collection of prescriptions of assistance in his work. Each recipe is labelled with the name of some symptom or disease for which the prescription is said to be suitable. It is bad enough to imagine that some doctors might wish for help in writing prescriptions, but to attach such labels as these shows that the editor is incapable of differentiating between the prescribing of the empirical pharmacist and that of the qualified medical man.

Elementary Bacteriology and Proto-Zoology. By Herbert Fox, M.D. (London: J. and A. Churchill. 1913. Pp. 237. Price 6s. 6d. net.)

This book contains a brief outline of the present state of our knowledge concerning pathogenic micro-organisms, together with a few facts showing how that knowledge is now being utilised to fight disease. It does not pretend to be anything more than an introduction to the subject, suitable for those who have had no previous experience or instruction on the subject. Within these limitations the book succeeds admirably, and should be extremely useful to the layman anxious to know something about this complex subject or to the probationer nurse who hears so much in her work of the influence of micro-organisms. A comprehensive glossary of the technical terms used will help both these classes of readers to absorb Dr. Fox's instruction. The only complaint we should like to make is in respect of the price, which is quite unnecessarily high for an elementary book of this size and scope, and may possibly militate considerably against its circulation.

